# The Principles and Practice of Surgery

**Published:** 1852-09

**Authors:** 


ART. V —*The Principles and Practice of Surgery.* Illustrated by three hundred and sixteen engravings on wood. By WILLIAM PIRRIE, F. R. S. E., Regius Professor of Surgery in the Medical College and University of Aberdeen; Surgeon to the Royal Infirmary, etc., etc. Edited with additions by JOHN NEILL, M. D., Surgeon to the Pennsylvania Hospital, Demonstrator of Anatomy in the University of Pennsylvania, etc. Philadelphia: Blanchard & Lea. 1852. pp. 184, 8vo.

*Lectures on the Principles and Practice of Surgery.* By BRANSBY B. COOPER, F. R. S., Senior Surgeon to Guy's Hospital, etc. Phila. Blanchard & Lea. 1852. pp. 771, 8vo.

*The Principles of Surgery.* By JAMES MILLER, F. R. S. E , F. R. C. S. E., Author of a Treatise on the Practice of Surgery; Surgeon in Ordinary to the Queen for Scotland; Surgeon in Ordinary to his Royal Highness Prince Albert, for Scotland; Professor of Surgery in the University of Edinburgh; Senior Surgeon to the Royal Infirmary, etc., etc. Third American from the Second and Enlarged Edinburgh Edition. Illustrated by two hundred and forty engravings on wood. Revised, with additions by F. W. Sargent, M. D., Member of the College of Physicians of Philadelphia; Author of "Minor Surgery," etc. Philadelphia: Blanchard & Lea. 1852. pp. 751, 8vo.

Here are three volumes appearing near the same time, of nearly the same size, making in the aggregate 2,306 large octavo pages, devoted to a single branch of the profession, and sent forth in the best style of the enterprising publishers, to whom American physicians are so largely indebted. Who can doubt, then, that we are a reading profession? What publishers would venture upon the issue of so many works on the same subject, if not assured that there was a demand for them? We take the fact of the rapid sale of medical books to be indubitable proof that there is a growing taste for study in the profession of our country.

Two of these works cover the same ground, and may be viewed in the light of rivals. Pirrie's, like Cooper's, consists of Lectures delivered to his students, and possesses all the qualities of a text-book. In his preface he states, that it is "for the use of his students" that it has been prepared. Both works are eminently practical, and afford that compendious view of the subject which students require in a text-book. Miller's is devoted to the *Principles of Surgery*, and is worthy of the high reputation it has attained at home and in this country. Its aim is to attract to the science of surgery more attention than it has ordinarily received. It is a philosophical work, in which the facts of surgery are combined and classified under definite laws. In this character it prefers strong claims to the favor of the profession. Our impression is that as a manual for students, Pirrie's is the best work extant; while the name and long experience of Cooper will recommend his volume to teachers, students, and practitioners.

